# Value that industrial collaborations bring to research and education efforts in universities: perspective of a professor working in the field of development of therapeutic proteins

**DOI:** 10.1186/1479-7364-7-7

**Published:** 2013-03-05

**Authors:** John F Carpenter

**Affiliations:** 1 Department of Pharmaceutical Sciences, Center for Pharmaceutical Biotechnology, University of Colorado Anschutz Medical Campus, Aurora, CO, 80045, USA

## 

The development (“D” in “R&D”) component for therapeutic protein products includes efforts to understand and improve bioprocessing methods (e.g., filling pump operation for vials and syringes, freeze-drying to create more stable product), product degradation pathways, and rational means to improve protein stability, new analytical methods, drug delivery methods, and investigation of key product parameters affecting product safety and efficacy. Research in these areas occurs in pharmaceutical companies and in universities. Often in companies, the focus is on doing the work that is needed to get a given product into clinical trials and on the market, whereas in universities, the focus is usually on more fundamental, mechanistic understanding of key issues such as factors causing protein aggregation and how to characterize and minimize this type of degradation. Therefore, there are numerous natural, synergistic collaborations between pharmaceutical companies and academic researchers, which provide for more rapid advancement of the field and relevant education of students than if the two groups worked independently. There is a long tradition of such productive and valuable collaborations between industry scientists and professors in pharmaceutical sciences departments. For this commentary, based on my two decades of experience with such collaborations, I will describe my views on the advantages of such partnerships and some of the pitfalls.

To start with, it is important to emphasize that a productive academic lab can rarely function and thrive on industry funding alone. There is also a need for funding from foundations or federal agencies to broaden the scope of the work in the lab, as well as to provide more long-term financial support for the lab. Typically, pharmaceutical companies will commit informally to funding a given project for at least a few years, i.e., for the duration of the research for a thesis project. However, often the formal collaborative research agreement will cover 1 year, with the anticipation that the project will be renewed annually. On the other hand, usually the project is “pre-approved” before the formal project proposal is sent to the company. This situation arises because the company researchers initiate the discussion about a funded collaboration by contacting the professor about a research project area. During these discussions, the groups work together to formulate a mutually agreeable outline for the project, with the understanding that funding should be available from the company. Then, it is just a matter of putting together a few pages of the technical proposal and a budget and letting the contract negotiators for the respective organizations start their work (see below for more on some issues with contracts). Recently, however, many companies have had changes in management philosophy and budgeting approaches for outside collaborations that may necessitate review of proposals by a company committee. In some cases, this committee has simply to approve the overall scientific aspects of the project and the perceived value to the company. However, in other cases, such committees must rank order the proposals, with only the top ones getting funding. Therefore, prior to entering into serious discussions with a company about a potential collaborative funded project, it is critically important to understand exactly how that company handles project proposals and decisions for funding, including the timing for evaluation of a proposal and funding decisions.

Because funding from pharmaceutical companies can be a valuable source of support for university investigators, new faculty often asks how they can obtain this sort of grant. In my experience, the companies come to you, based on peer-reviewed papers they have read of yours in areas of interest. Occasionally, the initial contact from company scientists can occur at a conference when the university lab is presenting the newest work. The key point is that most often, the company seeks out the professor rather than putting out some general call for proposals. This is an important issue to consider in the evaluation of faculty. In some departments, funding from the industry used to be viewed as somehow second class compared to grants from federal or foundation sources. However, most academics now realize that industry funding is actually a sign of success and recognition in the field; otherwise, companies would not approach a professor about a collaborative project.

It is also important to emphasize that industrial collaborations have tremendous value to the university missions of research and education, well beyond the provision of funding for a lab or a program.

1. The collaborations allow both groups to work on new areas of critical importance to the advancement of the drug development field and human medicine. If the project did not meet these criteria, a pharmaceutical company would probably not fund it.

2. Professors and students (also including postdoctoral researchers) get an opportunity to work closely with outstanding scientists in the industry. The contributions of the industry scientists to the fundamental and theoretical aspects of a project are often important. Furthermore, their practical insights into key pharmaceutical development questions can help shape a project such that, in addition to advancing basic knowledge, the work can have immediate impact on current drug development problems in the field. Furthermore, industry scientists mentor the students and help them to prepare for a career in a pharmaceutical company or a university.

3. Students on the project may get to spend time working in the labs of the sponsoring pharmaceutical company, which is of great value in their training both for scientific growth and increasing understanding of the industrial research environment. In some cases, the industry lab will have instruments and/or processing equipment that are not available in universities, providing the student with unique opportunities in a research project. Also, the students get to participate in a truly multidisciplinary research with experts in areas such as pharmaceutical sciences, analytical chemistry, regulatory affairs, immunology, and chemical engineering.

4. For our specific area of research, the therapeutic proteins provided by the collaborating company are invaluable for several reasons. Obtaining gram quantities of highly purified protein(s) is obviously much preferred to the costs and time of expressing and purifying a protein in the university. Also, work on real drug development problems is not of nearly as much value if some model nondrug protein (e.g., lysozyme) is used. Moreover, industry scientists will often already have worked out the key degradation routes for a given protein and many of the appropriate analytical methods.

5. Many times, companies will bring to the attention of an academic researcher a new emerging problem facing the industry. For example, we were approached by a company that found specific types of filling pumps causing formation of visible particles in a monoclonal antibody product. Published research on filling pump-induced damage was unheard of at the time, and university labs were not focusing on this area. As a result of the collaboration, we established filling pump studies in the university lab that led to new mechanistic insights into particle generation, successful thesis projects, and publications. More recently, the problems associated with protein aggregation caused by silicone oil in prefilled syringes were brought to our attention by several industrial collaborators, each of whom was interesting in different mechanistic aspects of the process and rational routes to inhibit it. As result, new understanding was obtained, a few different thesis projects were funded, and several research papers were published.

6. Students learn the critically important context for their research and get plenty of attention at conferences, as well as job offers from the industry.

7. With time, some of these students work their way into positions in pharmaceutical companies such that they can collaborate with (and fund) their former thesis advisor on projects.

8. By working collaboratively, industry and academic researchers can solve real problems and quickly lead to improvements in the speed and efficiency of drug product development and to greater safety and efficacy of these products. Such results improve medical care and reduce costs of medicines.

Of course, there are also potential pitfalls with collaborative, sponsored projects with industry. However, with insight into the key issues, these can usually be avoided.

1. First and foremost, it is absolutely essential that there is a specific language in the sponsored research agreement stating that the results of the research can be presented at conferences and published. Often, the company will have a provision that requires that abstracts, conference presentations, and manuscripts be sent to the company for review (usually regarding potential patents or proprietary information) prior to submission. This requirement means that the academic lab must plan well in advance of conferences to have the abstract, and then the presentation, completed. If the company decides that something is patentable in the results, then public disclosure of the work could be delayed. This does not have to be too long; patent applications can be prepared relatively rapidly. However, on rare occasions, presentation of a student’s research at a conference has been blocked because of a patent application. Of course, the student can then present the work at a later conference.

2. Companies (and their lawyers!) do not like to have information made public about drug products currently in development. Therefore, it is helpful if the research is going to focus on a therapeutic protein provided by the company and a protein that is no longer being developed is chosen, e.g., a protein that did not progress in clinical trials. For publication, the name, and perhaps sequence, of the protein drug may be required by the journal, but there are exceptions. For example, many therapeutic proteins are monoclonal antibodies. When these are used as model proteins in studies, it may not be necessary to disclose the name or sequence in a research publication.

3. The ownership of any inventions (or other intellectual property) will also be an important point of discussion during the contract negotiations. In my experience, no matter what the starting points of the university and the company, the negotiations lead to the same conclusion in the contract. The language states that if only university researchers are inventors, then the invention belongs to the university and the company can have first rights to negotiate a license to the invention. If the inventors are only company employees, then the company owns the invention. If, however, both university and company employees are inventors (which is often the case when a project actually leads to an invention), then each side can use the technology, with the understanding that the company also could negotiate to obtain exclusive rights to the university's ownership claim.

Nowadays, most university contract and technology transfer offices are well experienced in such negotiations. Thus, the faculty member does not need to get involved in these affairs. However, in some instances, the sort of intellectual property terms described above cannot be agreed upon, and the faculty member's input may be needed. If the company wants more ownership rights than is typical but the work is of critical importance for the university lab, the faculty member may be able to push for the contract to be completed, but of course, these issues vary on a case-by-case basis.

4. Company priorities and/or personnel can change, so a project that was informally anticipated to run for at least a few years may be not be renewed after a given year of funding. Also, as profitability of drug companies decreases, the management may decide that it can't afford to fund external projects. In these cases, hopefully, other funding sources (e.g., federal grants or bridging funds from the university) will be available to support the affected student.

Overall, the advantages of collaborations with the pharmaceutical industry far outweigh the potential pitfalls for university researchers. In addition to supporting sponsored research agreements between professors' labs and companies, it is important for universities to pursue larger, long-term collaborations with pharmaceutical companies. University faculty and administrators should appreciate the value of all of these types of collaborations in the furtherance of the education and research missions of the university and to advancing medicine.

